# Group Refractive Index of Nanocrystalline Yttria-Stabilized Zirconia Transparent Cranial Implants

**DOI:** 10.3389/fbioe.2021.619686

**Published:** 2021-03-19

**Authors:** David L. Halaney, Nitesh Katta, Hamidreza Fallah, Guillermo Aguilar, Thomas E. Milner

**Affiliations:** ^1^Laboratory of Guillermo Aguilar, Department of Mechanical Engineering, University of California, Riverside, Riverside, CA, United States; ^2^Laboratory of Thomas Milner, Department of Biomedical Engineering, University of Texas, Austin, TX, United States; ^3^Department of Physics, University of Isfahan, Isfahan, Iran

**Keywords:** brain, chromatic dispersion, cranial implant, group refractive index, imaging, optical coherence tomography, window to the brain

## Abstract

Transparent “Window to the Brain” (WttB) cranial implants made from a biocompatible ceramic, nanocrystalline Yttria-Stabilized Zirconia (nc-YSZ), were recently reported. These reports demonstrated chronic brain imaging across the implants in mice using optical coherence tomography (OCT) and laser speckle imaging. However, optical properties of these transparent cranial implants are neither completely characterized nor completely understood. In this study, we measure optical properties of the implant using a swept source OCT system with a spectral range of 136 nm centered at 1,300 nm to characterize the group refractive index of the nc-YSZ window, over a narrow range of temperatures at which the implant may be used during imaging or therapy (20–43°C). Group refractive index was found to be 2.1–2.2 for OCT imaging over this temperature range. Chromatic dispersion for this spectral range was observed to vary over the sample, sometimes flipping signs between normal and anomalous dispersion. These properties of nc-YSZ should be considered when designing optical systems and procedures that propagate light through the window, and when interpreting OCT brain images acquired across the window.

## Introduction

Neurosurgeries often involve craniectomy (removal of a portion of the cranial bone) to gain access to the brain for therapy, followed by the placement of a cranial implant to replace the excised bone. Cranial implants are normally made from a variety of materials including metals, polymers, and ceramics, and provide mechanical protection to the underlying brain tissue (Bonda et al., 2015). To our knowledge, current cranial implants available to patients lack optical transparency which could allow for brain optical imaging or therapy without implant removal or additional open skull procedures. We recently introduced a novel optically transparent cranial implant made from a biocompatible ceramic, nanocrystalline Yttria-Stabilized Zirconia (nc-YSZ), which we refer to as the “Window to the Brain” (WttB) implant (Davoodzadeh et al., 2018, 2019a,b; Cano-Velázquez et al., 2019). We have demonstrated chronic brain imaging across this implant *in vivo* usingoptical coherence tomography (OCT) (Halaney et al., 2020). OCT is an imaging technique based on broadband near-infrared light which can penetrate into scattering media such as brain tissue underlying the WttB implant. However, optical properties of these transparent cranial implants are neither completely characterized nor completely understood. Fundamental optical properties of the implant such as the group refractive index and chromatic dispersion are important to consider when planning or designing time-based and/or multispectral imaging strategies across the window, and for correct interpretation of recorded brain images. The group refractive index describes the speed at which a light wavepacket travels through the window, and is important to consider when focusing light across the window as well as when interpreting OCT images, where on-axis dimensions of reconstructed images are determined by time-of-flight of the OCT wavepacket. Chromatic dispersion also effects the quality of images recorded with broadband light sources like the ones used to record OCT images. Characterizing variation of group refractive index and chromatic dispersion will allow development of dispersion compensation techniques for obtaining higher quality OCT images. Furthermore, design of other multispectral imaging strategies that utilize cranial implant windows will be impacted by the group refractive index and chromatic dispersion.

Methods to measure refractive index include non-interferometric methods and interferometric methods. Non-interferometric methods include the use of index matching liquids, and refractometers. Index matching liquids are not ideal for determining the refractive index of nc-YSZ, which has lower transparency in the visible range and enhanced transparency in the near-infrared, because these liquids are typically designed for visible wavelengths and require switching the liquid until a near match is found, giving a rough estimate of the index of nc-YSZ. Refractometers require the thickness to be known at each location under interrogation, and due to the thin nature of our samples, this thickness measurement must be very precise (i.e., micron-scale) to yield an accurate index measurement. Because the samples we are measuring do not have perfect flatness nor perfectly uniform thickness, we needed a method which can simultaneously measure thickness and group refractive index at each location under interrogation on the sample. Of the interferometric methods available to measure refractive index, OCT is the most appropriate technique for our samples. Other interferometric methods are appropriate for highly transparent materials, whereas materials like transparent nc-YSZ exhibit some light scattering. While this scattering is enough to introduce artifacts in other methodologies, OCT has been used to measure the group refractive index of human skin, muscle and adipose tissue (Tearney et al., 1995), which is highly scattering, and thus is capable of accurately assessing the group refractive index of nc-YSZ. There are no other methods for these materials as precise as OCT, but because OCT is a broadband technique, we are able to assess group delay (GD) and group refractive index rather than phase index. Because OCT brain imaging is one of the most applicable imaging techniques for the Window to the Brain implant, group index over OCT wavelengths is highly applicable to envisioned clinical applications for the implant.

In this study, we measured optical properties of the transparent cranial implants using a swept source OCT system to characterize the group refractive index of the nc-YSZ window, over a narrow range of temperatures at which the implant may be used during imaging or therapy, ranging from room temperature (RT) to the point where thermal tissue damage begins to occur (20–43°C) (Yarmolenko et al., 2011). Additionally, chromatic dispersion of the OCT pulse was assessed.

## Materials and Methods

### Implant Fabrication and Preparation

**FIGURE 1:**
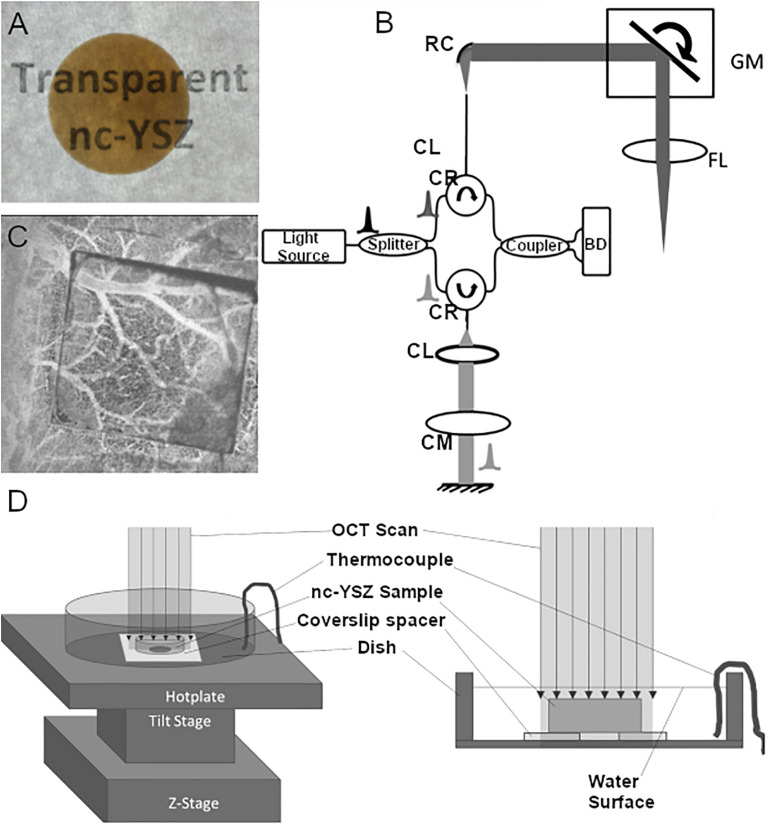
**(A)** A photograph of the nc-YSZ overlying the words “Transparent nc-YSZ” with backlighting. **(B)** Schematic of bench-top OCT system. Intensity OCT uses a Mach–Zehnder interferometer with circulators in the sample and reference paths (CR) using balanced detection (BD) and dispersion compensation (CM). Sample path delivery fiber APC; and reference path uses a reflective collimator (RC). Sample path uses galvo-mirrors (GM) placed at the back focal plane of the scanning lens (FL). **(C)** OCT angiography of mouse cerebral vasculature through a square nc-YSZ implant within a craniectomy, demonstrating the improved imaging across the implant compared to surrounding cranial bone. **(D)** Experimental setup for temperature-dependent measurements.

The transparent 8 mol% YO_1.5_ nc-YSZ WttB implant used in this study was fabricated from a precursor yttria-stabilized zirconia nanopowder (Tosoh USA, Inc., Grove City, OH, United States) densified into a bulk ceramic *via* current-activated pressure-assisted densification (CAPAD) as described previously (Garay, 2010). The resulting ceramic disk was 19 mm in diameter and 1 mm thick. The thickness was reduced further by polishing with 30 μm diamond slurry on an automatic polisher (Pace Technologies, Tucson, AZ, United States). The two faces were then polished using progressively finer abrasives (from 30 μm diamond slurry down to 0.2 μm colloidal silica slurry) to reduce light scattering by the implant surfaces and thus increase transparency. A photograph of the nc-YSZ overlying the words “transparent nc-YSZ” with back lighting is shown in Figure 1A.

### OCT Imaging

The OCT system used in this study utilized a swept-source, mode-locked laser (Axsun, Billerica, MA, United States) with central wavelength emission at 1,300 nm, 136 nm sweep and an A-scan-rate of 100 kHz (Figure 1B). The swept source output was coupled into a fiber-optic (SMF-28) Mach–Zehnder interferometer with pathlength and dispersion matched sample and reference arms. Sample path light was collimated (RC04, Thorlabs) and directed onto two galvanometer mirrors (GVS012, Thor Labs Inc.) positioned in a telecentric configuration with an aspheric ZnSe scanning lens (AR112-ZC-XWL-25-25, ISP Optics). An identical ZnSe lens was used in the reference path for dispersion compensation. Light backscattered from the sample and reflected from the reference mirror interfered and directed onto balanced detectors (BD) to obtain one-dimensional interferogram A-scans. Each one-dimensional scan (or A-line) collected contained 1,472 points (or pixels) to complete the interferogram. A pixel in the depth dimension was determined to correspond to a real thickness in air of 6.19 μm. Orthogonal scanning galvanometer mirrors in the scan head allowed for recording two-dimensional images (or B-scans where each B-scan consisted of 512 A-lines). Figure 1C shows an example of OCT angiography of mouse cerebral vasculature through a square nc-YSZ implant within a craniectomy, demonstrating the improved imaging across the implant compared to surrounding cranial bone.

### Experimental Setup for Temperature-Dependent Measurements

**FIGURE 2:**
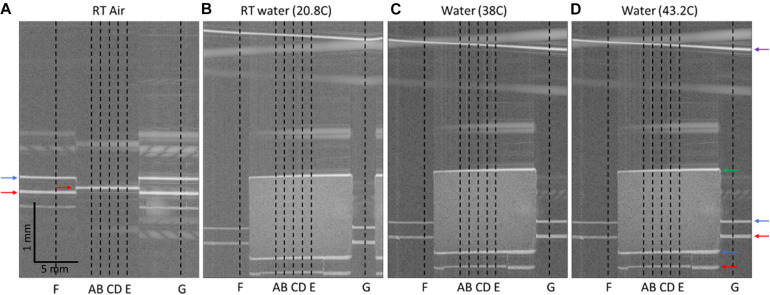
OCT B-Scans of nc-YSZ sample, with locations of interest identified. **(A)** Spacer and dish imaged in air at room temperature, **(B)** nc-YSZ sample imaged atop spacer and dish in water at room temperature (20.8°C), **(C)** nc-YSZ imaged in water at 38°C, **(D)** nc-YSZ imaged in water at 43.2°C. Locations A, B, C, D, and E are used to analyze nc-YSZ optical properties, while F and G are used to measure the real vertical displacement of the experimental setup within the imaging field. Horizontal arrows highlight relevant surfaces (red = dish; blue = spacer top/nc-YSZ bottom; green = nc-YSZ top; purple = water surface).

The OCT imaging head was placed over a glass dish containing a 100 μm coverslip spacer with hole in the center and secured in place with epoxy. The refractive index of the coverslip spacer (made of borosilicate glass) is 1.504 at central wavelength of OCT at 1.31 μm. The glass dish was placed atop a temperature adjustable plate on a tilt and *z*-stage, allowing for the dish to be positioned with normal incidence to the OCT beam (Figure 1D). A baseline image was acquired, showing the vertical height of the dish and spacer when imaged in air (Figure 2A). In Figure 2A, the dish surface appears displaced when imaged through the coverslip spacer compared to when imaged through the hole in the center of the spacer, due to the refraction of light in the coverslip spacer (red arrows), although this surface is in fact continuous and flat. Next, the nc-YSZ sample was carefully placed atop the spacer, and water was slowly added to submerge the sample. A thermocouple was used to measure the water temperature as 20.8°C. A RT image was acquired, showing the apparent displacement of the spacer and dish due to non-unity group refractive index of water (at locations F and G) or due to the nc-YSZ and water (at locations A, B, C, D, and E) (Figure 2B). Weight of the water also caused a real downward displacement of the dish due to compression of the temperature adjustable plate. The water temperature was increased by heating the temperature adjustable plate to 38°C (near body temperature). After the temperature stabilized, an OCT image was acquired. Displacement in the image was observed to change relative to the RT image due to several factors. The increased temperature changes the group refractive index of the water, and potentially of the nc-YSZ. Evaporation of the water during the heating to 38°C decreased the height of the air-water interface as well as decreasing the real displacement of the temperature adjustable plate *via* compression. The temperature adjustable plate also undergoes thermal expansion during heating, translating the dish upward in the image. Finally, the temperature was increased further, to 43.2°C. This temperature is near the upper limit with which the implant should be used *in vivo*. Higher temperatures were not attempted, as turbulence of the water became a confounding factor at temperatures above 45°C. Similar to the case with 38°C, the displacement in the image was observed to change relative to images recorded at lower temperatures due to the same factors discussed above.

### Group Refractive Index of Water

Refractive index of water was calculated using reference data (Thormählen et al., 1985), and assuming 1.0221 bar atmospheric pressure. Refractive index values were calculated for several wavelengths near the central OCT wavelength of 1,300 nm for each temperature of interest, and converted to group refractive index using Equation 1 (Paschotta, 2021):

(1) $$n_{g}=n-\lambda_{0}\cdot \left(d\,n/d\,\lambda_{0}\right)$$

where *n*_*g*_ is the group refractive index, *n* is the phase index of wavelength λ, and λ_0_ is the central OCT wavelength of 1,300 nm. Calculated group refractive index values of water are 1.337, 1.339, and 1.340 at 20.8, 38, and 43.2°C, respectively.

### Method 1: Image-Based Analysis (Group Refractive Index and Sample Thickness)

Using ImageJ, distances within the image were quantified (in microns and pixels). At locations of interest (A, B, C, D, E, F, and G in Figure 2), distances were measured from the top of the image to the following features: top surface of spacer, bottom of dish, top and bottom surfaces of nc-YSZ sample, and surface of water (see Figure 2). From these measurements, and group refractive index values of water at 20.8, 38, and 43.2°C, it is possible to separate the total displacement in the image Δ*_total_* into apparent displacement Δ*_*apparent*_* of the dish bottom and/or spacer (due to non-unity group refractive index of water and nc-YSZ) and the real displacement Δ*_real_* of the setup (due to compression and/or expansion of the temperature adjustable plate), using Equations 2, 3:

(2) $${△}_{\text{total}}={△}_{\text{apparent}}+{△}_{\text{real}}$$

(3) $${△}_{\text{apparent}}=T-d=T-\frac{T}{n\,g}$$

Where *T* = optical thickness, *d* = real thickness, and *ng* = group refractive index. First, the real displacement Δ*_*real*_* of the setup at each temperature is found using the total displacement Δ*_*total*_* of the spacer relative to the baseline image at locations F and G, where Δ*_*apparent*_* is caused by *ng*_*water*_ only. Combining Equations 2, 3, Equation 4:

(4) $${△}_{\text{real}}={△}_{\text{total}}-\left(T_{w\,a\,t\,e\,r}-\frac{T_{w\,a\,t\,e\,r}}{n\,g_{w\,a\,t\,e\,r}}\right)$$

The RT image had a real downward displacement Δ*_*real*_* = 4 px (∼25 μm) relative to the baseline image in air. This is due to the weight of the water compressing the temperature adjustable plate. The 38°C image had a real upward displacement Δ*_*real*_* = 2.5 px (∼15 μm) relative to the baseline image in air. This is due to thermal expansion of the temperature adjustable plate and experimental setup, as well as evaporation of some of the water compared to the RT image. The 43.2°C image had a real upward displacement Δ*_*real*_* = 4 px (∼25 μm) relative to the baseline image in air, due to thermal expansion of the temperature adjustable plate and experimental setup, as well as water evaporation compared to the RT and 38°C images.

Subtracting these real displacements from the total displacement Δ*_*total*_* in the images (using Equation 2) yields the apparent displacement within the image. To calculate the group refractive index of nc-YSZ from the apparent displacement, the method is the same as that used to compute real displacement Δ*_*real*_* above, except it is applied at locations A, B, C, D, and E, where the apparent displacement Δ*_*apparent*_* is due to non-unity group refractive index of water and nc-YSZ, using Equation 5:

(5) $$\begin{array}{c} {△}_{\text{apparent}}=\left(T_{\text{water}}-d_{\text{water}}\right)+\left(T_{n\,c-Y\,S\,Z}-d_{n\,c-Y\,S\,Z}\right)\\ =\left(T_{\text{water}}-\frac{T_{w\,a\,t\,e\,r}}{n\,g_{w\,a\,t\,e\,r}}\right)+\left(T_{n\,c-Y\,S\,Z}-\frac{T_{n\,c-Y\,S\,Z}}{n\,g_{n\,c-Y\,S\,Z}}\right) \end{array}$$

### Method 2: Spectral Phase Function Based Analysis (Group Delay and Group Delay Dispersion)

The second method of analysis uses the spectral phase function to calculate group refractive index and chromatic dispersion (Walmsley et al., 2001). The spectral phase function describes the relationship between the optical frequencies in the OCT pulse and the difference in phase for each frequency returning from the top and bottom surfaces of the nc-YSZ sample. The spectral phase function may be written as a Taylor series expanded about the central OCT optical frequency, Equation 6:

(6) $$\Phi \,\left(\nu \right)=\Phi^{\left(0\right)}+\Phi^{\left(1\right)}\,\left(\nu_{0}\right)\,{\left(\nu -\nu_{0}\right)}^{\prime }+\frac{1}{2}\,\Phi^{\left(2\right)}\,\left(\nu_{0}\right)\,{\left(\nu -\nu_{0}\right)}^{\prime \prime }+\frac{1}{6}\,\Phi^{\left(3\right)}\,\left(\nu_{0}\right)\,{\left(\nu -\nu_{0}\right)}^{\prime \prime \prime }.$$

Where Φ is phase, ν is optical frequency, *ν_0_* is the central OCT optical frequency, Φ^(0)^ is a common phase shift, Φ^(1)^(ν_0_) is the GD, Φ^(2)^(ν_0_) is the group delay dispersion (GDD), and higher order terms are higher order dispersion. Single A-lines were analyzed from the five locations of interest at the three temperatures. Each A-line was filtered using a Hilbert transform/narrowband phase-invariant spectral filter (Baumann et al., 2007) to isolate photons returning from the top and bottom surfaces of the sample (defined by full-width-half-maximum, FWHM, of the intensity peaks at the surfaces). These two phase functions correspond to the spectral phase functions of light returned from the sample’s top and bottom edges. Fitting this data with a polynomial curve can approximate the spectral phase function (Equation 6). 4th order polynomial fits of the optical phase vs. frequency data were performed in MATLAB (Mathworks, 2020). The curve fits were weighted by the normalized intensity spectrum of the OCT light source, and the high and low tails of the spectrum were trimmed by 200 pixels prior to the curve fitting to eliminate regions of low SNR and reduce the impact of noise. Root-mean-squared-error, RMSE, values were less than unity for all fits. The 1st order coefficient (Equation 6) of the spectral phase function approximation is the GD. The GD of each interface corresponds to the actual optical path length difference between the interface and the reference arm. A subtraction of these GDs for the top and bottom interfaces corresponds to the distance the light experienced within the sample (*T* = *ng*× *d*, Equation 2). The 2nd order coefficient (Equation 6) of the spectral phase function of the bottom surface is the approximation of half of the GDD the light experienced within the sample (from the top surface to the bottom surface of the sample).

## Results

Group refractive index of nc-YSZ was determined at each location and each temperature using the image-based analysis (Method 1) described above (Table 1). This method also allowed for determination of real sample thickness *d*_*nc*–_*_*YSZ*_* at each location and temperature, using the optical thickness *T* divided by the group refractive index *ng* (Equation 2) along with a calibration factor of 6.19 microns per pixel in the OCT image (Table 2). This method is based on whole pixels, and an error analysis shows that the measurement has a 2% error.

**TABLE 1:** Group refractive index of nc-YSZ measured at five locations and three temperatures, using an image-based approach.

ng_*nc–YSZ*_	20.8°C	38°C	43.2°C
A	2.159	2.159	2.168
B	2.164	2.150	2.165
C	2.176	2.164	2.159
D	2.176	2.174	2.159
E	2.181	2.173	2.176
Average	2.171	2.164	2.165
St. Dev	0.009	0.010	0.007

**TABLE 2:** Real thickness of nc-YSZ sample measured at the five locations and three temperatures, using an image-based approach.

d_*nc–YSZ*_	20.8°C	38°C	43.2°C
A	668 μm	668 μm	665 μm
B	669 μm	671 μm	663 μm
C	663 μm	669 μm	671 μm
D	666 μm	666 μm	674 μm
E	667 μm	669 μm	666 μm

Next, GD of the nc-YSZ sample was calculated using the spectral phase function approach (Method 2) described above. The 1st order coefficient of the spectral phase function approximation (Equation 6) is the GD. The GD for each location and temperature are shown in Table 3. From GD, group refractive index *ng* can be calculated using Equation 7 and sample thickness values *d*_*nc*–_*_*YSZ*_* obtained from Method 1 (Table 2):

**TABLE 3:** Group delay of nc-YSZ sample measured at the five locations and three temperatures.

GD	20.8°C	38°C	43.2°C
A	6.059e-11 s	6.034e-11 s	6.178e-11 s
B	6.059e-11 s	6.207e-11 s	6.116e-11 s
C	6.059e-11 s	6.126e-11 s	6.208e-11 s
D	6.125e-11 s	6.126e-11 s	6.118e-11 s
E	5.998e-11 s	6.147e-11 s	6.144e-11 s

(7) $$n\,g=\frac{G\,D\cdot c}{4\,\pi \cdot d}$$

Where *c* = 2.998e+8 m/s. Group refractive index values calculated using Equation 7 are shown in Table 4. Because this method uses sample thickness values determined from Method 1, it also has a 2% error in the calculated values of group refractive index, or an error of ∼0.043.

**TABLE 4:** Group refractive index of nc-YSZ sample calculated from group delay and sample thickness.

ng_*nc–YSZ*_	20.8°C	38°C	43.2°C
A	2.165	2.156	2.218
B	2.162	2.208	2.202
C	2.182	2.186	2.209
D	2.196	2.196	2.167
E	2.147	2.194	2.202
Average	2.170	2.188	2.200
St. Dev	0.019	0.019	0.019

The 2nd order coefficient of the spectral phase function approximation (Equation 6) is half of the GDD. The GDD for each location and temperature are provided in Table 5. Dividing the GDD by twice the sample thickness *d*_*nc*–_*_*YSZ*_* (round trip), the β-parameter of dispersion measured for the sample is shown in Table 6. Dispersion values varied between locations in the sample and between temperatures at a single location, even flipping sign between normal and anomalous dispersion. These values were reproducible by sequential A-lines at each location and temperature, causing us to believe this variability is not due to recording method or analysis.

**TABLE 5:** Group delay dispersion of nc-YSZ sample measured at the five locations and three temperatures.

GDD	20.8°C	38°C	43.2°C
A	−4.83e-27 s^2^	−3.16e-27 s^2^	1.40e-25 s^2^
B	−7.74e-27 s^2^	1.61e-25 s^2^	7.18e-26 s^2^
C	−4.71e-27 s^2^	8.25e-26 s^2^	1.59e-25 s^2^
D	8.02e-26 s^2^	8.26e-26 s^2^	8.01e-26 s^2^
E	−2.91e-25 s^2^	−1.26e-25 s^2^	7.89e-26 s^2^

**TABLE 6:** β-parameter of nc-YSZ sample calculated from GDD and sample thickness.

β	20.8°C	38°C	43.2°C
A	−3.61e-24 s^2^/m	−2.36e-24 s^2^/m	1.05e-22 s^2^/m
B	−5.79e-24 s^2^/m	1.20e-22 s^2^/m	5.41e-23 s^2^/m
C	−3.55e-24 s^2^/m	6.16e-23 s^2^/m	1.18e-22 s^2^/m
D	6.02e-23 s^2^/m	6.20e-23 s^2^/m	5.94e-23 s^2^/m
E	−2.18e-22 s^2^/m	−9.40e-23 s^2^/m	5.92e-23 s^2^/m

## Discussion

Group refractive index of the nc-YSZ window is important to consider when interpreting OCT images of underlying brain tissue, like those we reported previously (Halaney et al., 2020). Because OCT is a time-based imaging technique, on-axis dimensions in reconstructed OCT images depend on time-of-flight of the OCT pulse. When imaging across the WttB implant with a group refractive index of ∼2.1, the implant appears 2.1 times of its mechanical thickness, and displaces other features beneath the window downward in the image. This effect can be visualized in Figures 2B–D, where the coverslip spacer beneath the nc-YSZ implant is displaced downward and appears discontinuous with the parts of the coverslip spacer on either side of the implant (blue arrows). Knowing the group refractive index is also important for focusing light through the window onto underlying tissue. Changes in group refractive index between the different temperatures investigated in this study (20–43°C) were ≤0.036, and within the 2% uncertainty of the sample thickness measurement. This temperature stability is not surprising, since YSZ has high thermal stability (Ghosh et al., 2009) and stable broadband IR reflection at much greater temperatures than those used in this study (i.e., >1400°C) (Leib et al., 2016).

**FIGURE 3:**
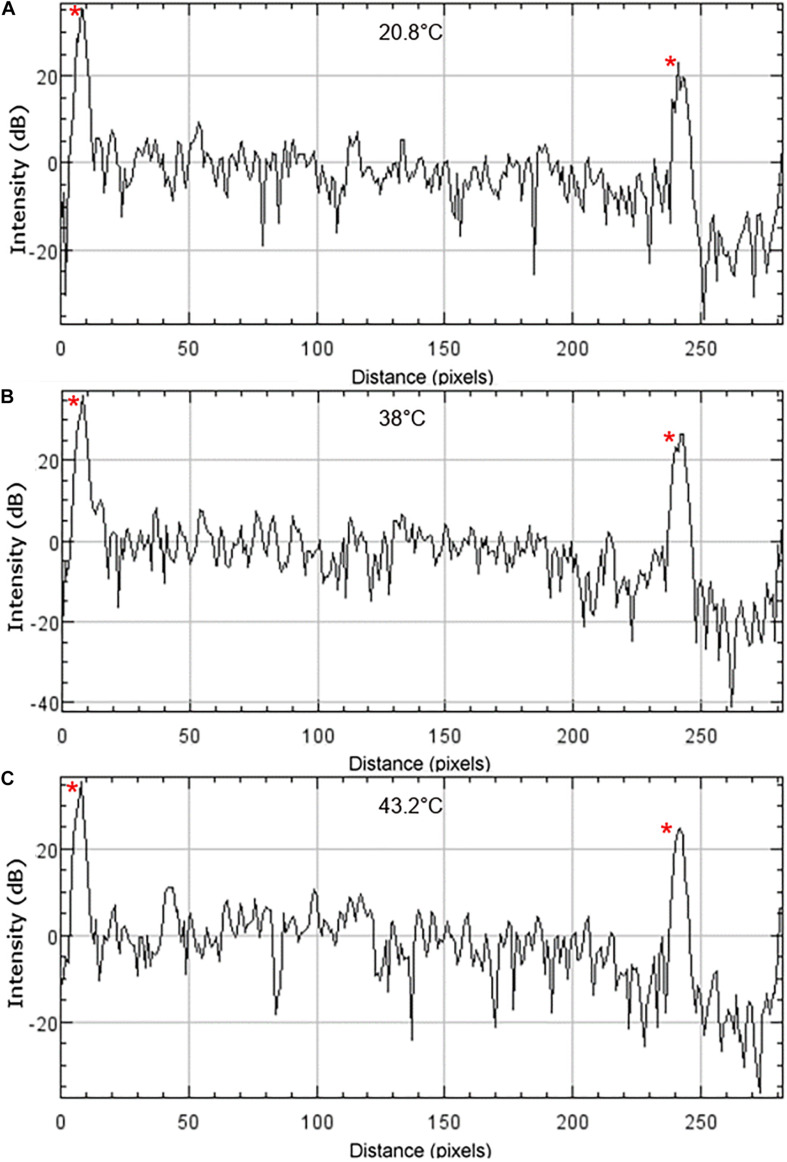
OCT intensity profiles of nc-YSZ sample at location E at **(A)** 20.8°C RT, **(B)** 38°C, and **(C)** 43.2°C. The upper and lower surfaces of the sample are visible as intensity spikes on the left and right side of the profiles, respectively, and are identified with red asterisks.

Chromatic dispersion values were found to vary across different sample locations and different temperatures at fixed locations, even flipping sign between normal and anomalous dispersion (Tables 5, 6). Despite this variability, individual measurements were reproducible by repeated A-lines at each fixed location and temperature. Thus, this variability may be a material property and not an artifact of data recording or analysis. The observed dispersion variation may be due to light scattering within the bulk of the sample at grain boundaries. Scattering within the sample is apparent in recorded OCT images shown in Figure 2, and is quantified in Figure 3 at location E for each temperature. This chromatic dispersion should be studied further, and will be an important consideration for any multispectral optical approaches that propagate light through the window where precise pulse duration needs to be maintained.

There were several limitations to the current study. As explained in the introduction, an additional method to validate the OCT findings was not conducted, due to the scattering properties of our samples and the need for simultaneous determination of the sample thickness at each location being interrogated. Additionally, while this measurement covered the wavelength range and temperature range anticipated for clinical applications of the implant with OCT brain imaging, wavelengths, and temperature effects outside of this range were not assessed. The values of group refractive index reported here are applicable to light with a spectral range of 136 nm and centered at 1,300 nm only.

## Conclusion

Nc-YSZ cranial implant windows have a group refractive index of 2.1–2.2 for OCT imaging with a spectral range of 136 nm centered at 1,300 nm at normal working implant temperatures (20–43°C). Chromatic dispersion for this spectral range was observed to vary over the sample, sometimes flipping signs between normal and anomalous dispersion. These properties of nc-YSZ should be considered when designing optical systems and procedures that propagate light through the window.

## Data Availability Statement

The raw data supporting the conclusions of this article will be made available by the authors, without undue reservation.

## Ethics Statement

The animal study was reviewed and approved by Institutional Animal Care and Use Committee, University of Texas at Austin.

## Author Contributions

DH, NK, and HF performed the OCT imaging and analysis. TM and GA provided guidance on study design and data interpretation. All authors contributed to manuscript revision, read, and approved the submitted version.

## Conflict of Interest

The authors declare that the research was conducted in the absence of any commercial or financial relationships that could be construed as a potential conflict of interest.
